# A Qualitative Exploration of Providers’ Approaches to Relational Harm Reduction in HIV Primary Care Settings

**DOI:** 10.21203/rs.3.rs-4172083/v1

**Published:** 2024-03-29

**Authors:** Emma Sophia Kay, Stephanie Creasy, Jessica Townsend, Mary Hawk

**Affiliations:** University of Alabama at Birmingham; University of Pittsburgh; University of Alabama at Birmingham; University of Pittsburgh

## Abstract

**Background:**

Structural harm reduction is an approach to care for people who use drugs (PWUD) that incorporates services and resources (e.g., naloxone, sterile syringes). As conceptualized in our previous research, harm reduction is also “relational,” encompassing a patient-provider relationship that is non-judgmental and respectful of patients’ autonomy. Little is known about providers’ knowledge or attitudes towards harm reduction beyond structural strategies, whose availability and legality vary across geographical settings. To operationalize how relational harm reduction is both characterized and employed in HIV care settings, where nearly half of patients have a diagnosed substance use disorder, we qualitatively explored providers’ knowledge of and use of harm reduction via individual in-depth interviews.

**Methods:**

Our study sample included three HIV clinics, one in Birmingham, Alabama (AL) and two in Pittsburgh, Pennsylvania (PA). We conducted individual interviews with n = 23 providers via Zoom, using a semi-structured interview guide to probe for questions around providers’ attitudes towards and experiences with providing care to PWH who use drugs and their knowledge of and attitudes towards relational and structural harm reduction. Data was analyzed in Dedoose using thematic analysis.

**Results:**

Qualitative analyses revealed three primary themes, including *Relational Harm Reduction in Practice, Not Harm Reduction, No Knowledge of Harm Reduction, and Harm Reduction Training*. Nearly all providers (n = 19, 83%) described a patient interaction or expressed a sentiment that corresponded with the principles of relational harm reduction. Yet, over half of participants (n = 14, 61%) used language to describe PWH who use drugs that was stigmatizing or described an interaction that was antithetical to the principles of relational harm reduction. Five providers, all from Birmingham, were unaware of the term ‘harm reduction.’ Few providers had any harm reduction training.

**Conclusion:**

Our findings suggest that relational harm reduction in HIV care settings is practiced along a continuum, and that a range of behaviors exist even within individual providers (e.g., used stigmatizing terms such as “addict” but also described patient interactions that reflected patients’ autonomy). Given that harm reduction is typically described as a structural approach, a broader definition of harm reduction that is not dependent on policy-dependent resources is needed.

## INTRODUCTION

An estimated 48% of people with HIV (PWH) in the United States (US) have a diagnosed substance use disorder (SUD) [1]. Compared with PWH who do not use drugs, PWH who use drugs experience lower rates of retention in HIV care[2, 3] and viral suppression [4–6], thereby increasing risk of morbidity and mortality[7, 8]. PWH who use drugs are also more likely to present to care with advanced HIV diagnosis[9, 10]. Moreover, injection drug use increases the risk of HIV acquisition and transmission, with an estimated 10% of new HIV diagnoses attributed to injection drug use in 2018[11].

Harm reduction is an approach to care for people who use drugs (PWUD) that incorporates not only services and resources (e.g., naloxone, sterile syringes, fentanyl test strips—structural harm reduction), but also patient-provider relationships that are non-judgmental and respectful of patients’ autonomy, defined as relational harm reduction[12]. Harm reduction is aimed at minimizing harm associated with drug use, rather than requiring abstinence. In a previous study, we outlined a set of six harm reduction principles in medical settings, which can be used to guide providers’ interactions with patients (citation redacted for peer review).

Yet, substance use-related stigma, particularly stigma enacted by providers and experienced in the healthcare setting, overwhelmingly contributes to disparate outcomes among PWUD. A recent editorial by the director of the National Institute on Drug Abuse (NIDA) urges healthcare providers to provide compassionate, non-stigmatizing care to PWUD, noting the alternative may exacerbate drug use[13]. Continued conflation of drug use as “abuse,” which implies that any drug use is wrong, pervades social messaging[14]. Indeed, research shows that providers are not immune from this social messaging, with some providers regarding PWUD as “criminal”[15]. To avoid experiences of stigma and discrimination when receiving health care services, PWUD may seek to avoid stigma by concealing their drug use from providers, minimizing symptoms of pain, and even delaying care altogether[16, 17]. PWUD may even avoid calling emergency medical services for fear of arrest[18]. These negative experiences in healthcare settings decrease trust in the medical system, raising risk of adverse health outcomes such as death from injection-related infections[17], relying on non-prescription medication to alleviate pain[19], and leaving the hospital against medical advice[20]. However, patients who feel respected by and trust their providers are more likely to experience positive health outcomes. For PWH, this translates into disclosing more information to and having a better relationship with providers,[21] better adherence to antiretroviral therapy (ART) [22–24], and high rates of retention in care[25, 26]. For PWUD, greater trust in their provider is associated with positive expectations for their interactions with their providers and is mediated by perceived provider support for harm reduction[27]. PWUD also cite the harm reduction principles of humanism, pragmatism, autonomy, individualism, incrementalism, and autonomy without termination[12], as well as ongoing support, reliability, and provider expertise in treating substance use disorder[28], as cornerstones of strong patient-provider relationships.

To better serve the needs of PWUD, scholars and providers have recommended integrating harm reduction into primary care and other settings that do not explicitly serve PWUD and have recognized the importance of the patient-provider relationship as a form of harm reduction[29]. Indeed, harm reduction has been recognized as one of the key components of the US Department of Health and Human Services’ Overdose Prevention Strategy[30], and the Health and Medicine panel of the National Academy of Sciences, Engineering and Medicine has recommended incorporating harm reduction strategies into infectious disease and opioid use disorder care[31]. Since the elimination of the X-Waiver in 2023, any provider can prescribe buprenorphine without having to register with the Drug Enforcement Administration[32], a requirement that was previously noted as a significant barrier to prescribing medication for opioid use disorder (MOUD) [33]. Yet, despite the importance of integrating harm reduction principles into health care settings, little is known about the extent to which this is done in healthcare settings outside of specialty care for PWUD or about providers’ knowledge and use of harm reduction beyond physicians. A previous scoping review focusing on harm reduction for people who use opioids identified 25 studies that examined physicians’ knowledge and perceptions of harm reduction for people who use opioids[34]. Knowledge gaps include those related to prescribing medication for opioid use disorder and using naloxone, and uncertainly about their legality. Physicians’ perceptions of harm reduction highlighted the prevalence of stigma and concerns about medication diversion[34]. Finally, the scoping review revealed system and institutional barriers to the provision of high-quality care for PWUD, such as those related to insurance coverage, reimbursement, and organizational policies. Similarly, a survey of Veterans Affairs providers identified low levels of knowledge regarding use of naloxone[35].

Despite the high SUD comorbidity rate among PWH, no extant research, to our knowledge, has focused on HIV providers’ knowledge of or attitudes towards harm reduction. Moreover, little is known about medical providers’ knowledge of harm reduction beyond structural strategies like MOUD and naloxone. Given the variability of these services across different settings and political contexts, the role of relational harm reduction in medical settings is important to understand.

Thus, in the current paper, we qualitatively explore providers’ knowledge of and use of harm reduction via individual in-depth interviews, to operationalize how relational harm reduction is both characterized and employed in HIV care settings The current work is part of our larger study aimed at developing a harm reduction intervention for PWH who use drugs (citation redacted for peer review).

## METHODS

### Sample and Recruitment

Our study sample included three HIV clinics, one in Birmingham, Alabama (AL) and two in Pittsburgh, Pennsylvania (PA). We leveraged internal electronic messaging used by each site to disseminate information about the provider interviews. IRB-approved recruitment messaging and a confidential link to the REDCap survey were included in the email messages, which were sent by site-level champions. Providers were eligible if they (1) worked at one of these sites for at least one year and (2) provided service or care to PWH or PWUD at high risk for HIV acquisition. Since modern HIV care is akin to a medical home model with coordinated medical and social services [36, 37], we defined “provider” as anyone who had face-to-face contact with patients, including pharmacists, social service providers, medical service providers, and administrative service providers. We also defined “drugs” as inclusive of illicit drugs and prescription drugs used in ways other than they were prescribed; we did not include alcohol or marijuana use, as these substances have been shown to carry less stigma than prescription drug misuse or use of drugs that are nationally criminalized[38, 39].

Individual interviews with n = 23 providers were conducted over a four-month period between November 2022 and March 2023. Interviews lasted between 30 and 60 minutes (average = 45 minutes). To maximize availability of the five study team members who led the qualitative interviews across Birmingham and Pittsburgh, interviews were conducted over HIPAA-compliant Zoom. Each of these study team members, including the PIs, three Co-Is, and a study coordinator, provided their availability on Microsoft Bookings. Interested providers could then sign up for an available timeslot with a particular interviewer, thereby streamlining the recruitment process.

### Data Collection

We used a semi-structured interview guide to explore providers’ attitudes towards and experiences with providing care to PWH who use drugs and their knowledge of and attitudes towards relational and structural harm reduction. We collected demographic information around participants’ racial and ethnic identity and gender identity, job title, and years of practice, including years specifically devoted to working with PWH, which we used to characterize the participant population in aggregate. Interviews were audio-recorded with participant permission and professionally transcribed verbatim. All identifying information was removed from study transcripts; each transcript was labeled with a numerical subject identification number and the information linking subject identification numbers with names was kept separate from the research records. All study activities were approved by the [name redacted] IRB.

### Analysis

Deidentified transcript data were uploaded into Dedoose[40] for analysis. We used Braun and Clark’s six-step process for thematic analysis[41, 42] to code the data. One of the PIs (first author) read through each of the transcripts and familiarized themselves with the data. They reviewed field notes composed by study team members who conducted the interviews, which provided valuable critical reflection and interviewer feedback to inform analysis[43]. Then, this PI, in addition to four other members of the study team with expertise in qualitative analysis, independently coded three transcripts to identify broad themes. This team of five then met to discuss initial codes and resolve any discrepancies.

This list of initial codes was used to create a coding framework. The first author and three other team members coded the remaining transcripts using this framework, meeting every two weeks and iteratively adding sub-codes and modifying the codebook using processes of adjudication. Five transcripts were double-coded (23%) and compared for consistency, following scholars’ recommendation to double-code between 10–25% of transcripts[44]. The final set of codes was combined into themes with the input of the full study team.

## RESULTS

Twelve interviews were completed with providers in Birmingham, and 11 were completed with providers in Pittsburgh. Table 1 provides an overview of self-reported provider characteristics.

Qualitative analyses revealed three primary themes and several subthemes characterizing a range of relational harm reduction knowledge and practice. Primary themes included *Relational Harm Reduction in Practice, Not Harm Reduction, and No Knowledge of Harm Reduction*. An additional interrelated them on *Harm Reduction Training* also emerged.

### Relational Harm Reduction in Practice

Nearly all providers (n = 19, 83%) described a patient interaction or expressed a sentiment that corresponded with the principles of relational harm reduction. Primary subthemes, each of which corresponded to one or more harm reduction principles (listed in paratheses beside each subtheme), included *Meeting Patients Where They Are, Knowing Patients as Humans, Open Communication*, and *Always Having the Door Open*. See Table 2 for a list of the relational harm reduction principles and their definitions.

### Meeting Patients Where They Are (Individualism; Autonomy)

Some providers described how they allowed patients to guide and lead their interactions rather than imposing a set agenda, as in the case of the following provider:

I believe we do harm reduction every day…not showing or casting any kind of judgment on a patient, meeting them where they are and just listening, just trying to guide them through where you can and where they allow you to. (PA.7)

This sentiment was expressed across provider types in ways that reflected their respective job responsibilities. Social service providers described how they helped patients access wraparound social services like housing or how they used motivational interviewing to “meet patients where they’re at,” while medical providers focused on aspects of clinical care:

I’m pretty good at kind of coaching patients through how to do it at home and, you know, they have good contact information with us should anything happen, should they need to talk to us. I kinda go over the different induction options with them, whether they wanna do traditional high dose or, um, low dose induction, and kind of just figure out what works for the patient. (PA.1)

### Knowing Patients as Humans (Humanism)

Most providers emphasized the importance of humanizing their patients and getting to know them as people. This involved asking patients about their lives outside of their health condition(s) and creating a space where patients felt comfortable sharing personal details. An AL provider discussed how they get to “know their aunties and their dogs and their cats,” while a PA provider shared how he starts clinical visits by catching up:

You know, my first thing was, you know, “How have you been doing...We’ll get to your vitals and, you know, going over your medications, but how are you? Um, you know, how was your week?” You know, and it’s just, like, starting off like, “…I wanna make sure you’re okay.” (PA.2)

Some providers described this humanizing approach as “more important than what ailments our patients have” and shared that their patients appreciated being treated as a person rather than “number on a page.” Providers also personally benefited from having meaningful relationships with patients. An AL provider described their patients as “family members,” while a PA provider described their relationships with patient as a “privilege”:

When you see someone periodically every four to six months for years and you’re walking a journey with them… I consider a privilege to do. It’s, um, it, it’s very rewarding, and …I think it’s mutual. (PA.4)

Another PA provider highlighted the importance of maintaining long-term relationships with patients, which allows them to trust and “start opening up.” Finally, one provider expertly summarized the relationship between relational and structural harm reduction and the reason why both are important in caring for PWUD:

We wanna make sure you have a relationship with people that you’re taking care of and it not be as transactional. However, a lot of harm reduction transactions, especially in the beginning, can be very transactional. Like, they might come in, just want syringes, and wanna leave. And they don’t necessarily wanna hear anything you have to say. Um, and that can take time. And if that’s all that ever happens, then fine. You’re still making a difference in their life because they’re somewhere safe that they feel like they can go and get safe things that they need. Um, so it can start transactional, but become very relational and, like, become a long-lasting relationship. And you can help people through ups and downs. (PA.9)

### Open Communication (Incrementalism; Pragmatism)

Providers underscored the importance of creating an environment of mutual trust with patients where they felt comfortable disclosing drug use, thereby allowing providers to give patients more tailored care:

Um, you know, it should always be a no-judgment zone. You know, don’t pass judgments. You know, yeah, we want to know what’s going on with you, and it’s important for you to be honest because it only will allow us to, you know, individualize your care, um, based off of what you’re dealing with. (PA.2)

Providers also recognized the multiple and complex challenges that some patients faced and acted like “cheerleaders” whenever patients experienced setbacks:

### Interviewer

What if they don’t make any progress at all or go backwards?

### Provider

So I have some patients that are like that, and they’re like, “I almost didn’t come into my visit today because I was so upset that I have made no progress.” And I was– and then I just thank them for being honest, and that, you know, we can try again. …and then I talk about like, “Why did you not make progress? Like, was there a reason?” And they’re, you know, their mom died, and they got evicted. You know, and it’s like, “Okay. You had other things going on is reasonable that you were stressed out and that wasn’t the first thing on your list.” (AL3)

### Always Having the Door Open (Accountability Without Termination)

Providers discussed the importance of maintaining continuity of care with patients and not terminating anyone from care for continued drug use. An AL provider shared how she was committed to “[getting] through this together” with patients:

One thing that I do like about our [Clinic] is they will, like, never terminate anyone. So a person– you know, like, they have all these other drugs in their subsystem, but they’re still able to come back. … I’ve had so many patients who’ve gone through treatment and relapsed again. I’m like, “It’s okay. You know, we’ll get through it together. You know, we can always try again. Like, you’re still here, and that’s the point. (AL.5)

Providers emphasized that their goal was to help patients, rather than “punish [them] for normal human behavior.” The only instances in which patients were terminated from care involved threats of violence.

### Not Harm Reduction

Over half of participants (n = 14, 61%) used language to describe PWH who use drugs that was stigmatizing or described an interaction that was antithetical to the six principles of relational harm reduction. These participants included providers across AL and PA and encompassed a range of provider types. Several subthemes also emerged, including *Substance Use Stigma* and *Characterizing Abstinence as the End Goal for All PWUD*.

### Substance Use Stigma

Substance use stigma was evident among some of the providers who used stigmatizing terms like “addict” or who characterized PWUD negatively. For some providers, stigma was evident in the way they couched PWUD as being “less than” patients who do not use drugs. This distinction often had moralistic or paternalistic tones and included framing abstinence as “bettering yourself,” a “[straight and] narrow path,” becoming a “productive member of society,” etc., without consideration of around whether abstinence aligned with the patient’s own goals. Other providers emphasized the importance of compliance rather than patient autonomy, as in a PA provider who described what a good interaction with a patient would look like: “Um, just breaking a barrier to let them know that everything can be okay and will be okay as long as you are compliant, you listen, and do what’s asked of you to be done.” (PA.7) In these examples, substance use stigma is evident in the ways that providers devalue patients’ autonomy, implying that PWUD are not capable or worthy of setting their own goals, Stigma also impacted providers’ behaviors. For example, a PA provider described how she had witnessed other providers at their clinic deny giving pain medication to PWUD due to assumptions they would sell it.

### Characterizing Abstinence as the End Goal for All PWUD

A key tenet of harm reduction is an understanding that patients’ goals might not reflect that of providers’, and that abstinence may not be the goal for all PWUD. However, some providers described harm reduction as a kind of stepping-stone on the way to abstinence, implying that abstinence is the only acceptable outcome for patients:

I mean, harm reduction is a part of soberness. I feel like it’s the first step to getting people to a point where they’re willing to contemplate changing, and making them more aware of what they’re actually doing...because in order to practice harm reduction, you have to be aware that you need to. And if you’re aware that you need to, then you’re aware of what you’re doing, and you’re aware that it could cause problems. And if you get to that point, then you might be willing to talk about further initiatives for change. (AL.3)

Other providers favored abstinence even more strongly, framing harm reduction as “enabling” drug use:

The only time I have a problem with [harm reduction] is when I think people are, like, really taking advantage of it, that they’re just manipulating the system because they know we’re harm reduction. I don’t see that in the majority of our patients, but, you know, I definitely have seen it a few times, and it makes me, like, upset because I’m like, “We’re doing everything we can do for you, and you’re still, like, either not listening or not, like, doing what you, you need to be doing.” (PA.8)

An abstinence stance was also seen in providers who regarded a prescription for MOUD as “being rewarded for doing drugs” or as a preferably time-limited medication rather than “[taking] Suboxone for the rest of your life.”

### No Knowledge of Harm Reduction

Though reflecting a minority of participants, several providers (n = 5, 22%; all from Birmingham) were unfamiliar with the term “harm reduction.” However, after being given a standardized definition from the interviewer, some providers were able to relate it back to their work. For example, a dietician shared how she could use harm reduction in her practice:

I kind of use harm reduction in nutrition because I hope to help people balance, right? So people who don’t want to give up sodas, for example, maybe we can think of something else they could do to, to, you know, reduce that harm, like, walking more, whatever. (AL.3)

Similarly, an administrative provider working with dental patients identified how harm reduction could relate to dental care for PWUD, noting that patients could decrease drug use to protect their dental health.

### Harm Reduction Training

Few providers in our study had formal training in harm reduction, regardless of their job title. Most had little experience working with PWUD prior to their current roles and were not familiar with harm reduction outside of structural services, despite many providers describing interactions with patients that aligned with one or more principles of relational harm reduction.

Overall, providers in AL had less exposure to harm reduction than providers in PA. Primary sources of information about harm reduction came from webinars, conferences, and social media. As one provider noted, X (formerly known as Twitter) was a primary source of information: “Yeah, a lot of it has been me, um, just reading on my own and following– I mean, frankly, a lot of it I get through Twitter. A lot of the people I follow on Twitter are harm reductionists.” AL providers also discussed the importance of exposing more medical students to harm reduction to “destigmatize” working with PWUD:

And you’re right, it’s not really trained. At least I wasn’t trained, in the way that I practice now, when I was in residency or fellowship. There was no substance use clinic that I could shadow at or kind of rotate through when I was in training. And I wish there was. And we certainly have the fellows come and experience [Clinic] now when we have residents and PA students. We try to get as many learners as possible for this reason, that we want to open their eyes…to this whole kind of population in need that is, and it’s really satisfying work. (AL.5)

Providers in PA described receiving structural harm reduction education at multiple levels of influence, including the healthcare network, organization, and patient levels. One provider described how their work with patients made her a better harm reductionist and demonstrated use of both structural and relational harm reduction:

Just constantly be getting feedback from the people you’re helping and what they think about something as simple as, like, really, “I hate this brand of syringes. Like, they keep breaking. They don’t [plunge], and, like, that kinda feedback. Really trying to make it comfortable for everybody and just learn as you go. (PA.9)

Another PA provider also characterized harm reduction as being innate rather than something that needed to be taught:

I guess [harm reduction]’s not something that can be taught…it’s all about, you know, caring about the next person, no matter what they’re dealing with, wanting to see everybody succeed, even, like, professionally. (PA.2)

## DISCUSSION

These qualitative findings reveal the extent to which relational harm reduction exists as a continuum in HIV care settings. Harm reduction can even occur along a continuum for a single provider, as evidenced by individual providers in our study who used stigmatizing terms such as “addict” or “drug abuse,” for example, but who also described patient interactions that reflected principles of relational harm reduction. Interestingly, we did not identify any place- or provider role-based trends in providers’ use of relational harm reduction, suggesting that this continuum may exist despite variation in legality of harm reduction services (e.g., syringe services not legal in AL but are legal in Pittsburgh, PA).

Our study also identified the need for more relational harm reduction training. Few providers had received any formal education in either relational or structural harm reduction. AL providers were primarily self-taught and discussed the paucity of training, which is likely a result of having fewer resources for PWUD, including a lack of legalized sterile syringe programs (SSPs) [45]. However, even providers in Pittsburgh, where SSPs operate legally[46], primarily learned on-the-job rather than via a formal training program. Interestingly, while PA providers spoke about learning of the ethos of harm reduction from their clients, AL providers did not identify clients as a source of harm reduction knowledge. Another difference between the two groups of providers was that only PA providers discussed harm reduction as an innate method of caring for PWUD. While it is difficult to speculate on the reasons for these setting-based differences that are not a direct reflection of policy context, it may be that policy-level differences had a downstream effect, such that AL providers felt less empowered or confident as harm reductionists given more limited resources. However, some providers who stated that they were not familiar with or had not been trained in harm reduction were able to provide examples of relational harm reduction in practice, suggesting that some providers may be practicing harm reduction without recognizing it as such. Given that harm reduction is typically described as a structural approach, a broader definition of harm reduction that is not dependent on policy-dependent resources is needed.

Providers emphasized the importance of getting to know their patients as human beings beyond their health diagnoses (humanism). This led to enhanced patient comfort and a sense of fulfillment for providers. A personalized relationship has been identified as one of the strongest independent predictors of adherence to antiretroviral therapy for PWH[22]. The importance of the patient-provider relationship and the significance of providers earning patients’ trust in harm reduction and substance use treatment settings has also been recognized in extant literature[47–49]. Yet, less is known about the extent to which humanism impacts substance use-related health outcomes. Additional research is needed to explore this potential relationship.

Harm reduction does not preclude abstinence and may be a treatment goal for some PWUD. The emphasis on abstinence for all PWUD among providers in our study, and even the suggestion that MOUD should be a time-limited healthcare service despite the wide evidence base to the contrary[50], is reflective of a larger cultural emphasis on sobriety and the widespread criminalization of drug use. Scholars have noted that moralism pervades anti-harm reductionist views, and that, despite an economic and medical evidence base supporting harm reduction[51], a belief that drug use is “immoral” diminishes support for harm reduction policies and programs[52]. Favoring abstinence may also decrease support for harm reduction programs[53]. Yet, as our study demonstrates, support for harm reduction and attitudes towards abstinence may not always be linearly related, and harm reduction often exists on a continuum. Even organizations that officially incorporate harm reduction may still favor abstinence and stigmatize people who are actively using drugs. For example, a qualitative study of staff and residents at a housing first program described how abstinence was characterized as “improving one’s life” and emphasized the importance of “getting clean”[54]. Participants also spoke about the disconnect between policy and practice, in which abstinence was not required for program entry but substance use onsite was not tolerated and could lead to dismissal[54]. Similarly, while all HIV clinics included in our study directly provided or referred patients to harm reduction services, abstinence was prioritized among some of the providers and clearly pervaded their interactions with patients.

### Limitations

These qualitative findings reflect the perspective of HIV providers in Birmingham, AL and Pittsburgh, PA, and may not reflect the attitudes of providers who work outside of HIV clinics or elsewhere in the United States. Recruitment language shared with providers stated that “the aim of this study is to understand the ways that harm reduction care and stigma experienced in healthcare settings affect clinical outcomes for people living with HIV who use drugs.” As a result, providers who elected to participate in the interviews may have been more knowledgeable about harm reduction than those who did not. These perspectives may therefore not be representative of those of providers less familiar with harm reduction work. However, results demonstrated a wide range of harm reduction approaches and variable familiarity with the concept, suggesting that our sample was fairly heterogeneous.

### Conclusion

Our study is the first, to our knowledge, that explores how HIV providers utilize relational harm reduction in HIV primary care settings. Our findings suggest that relational harm reduction in these settings is practiced along a continuum. Some providers were experienced in integrating relational harm reduction into their interactions with patients, while several providers were entirely unaware of harm reduction. Interestingly, we also found that even strong harm reductionists shared sentiments or used language in opposition to harm reduction principles, suggesting that even experienced providers could benefit from additional training. Given the health benefits associated with harm reduction care, additional research is needed to identify ways to strengthen harm reduction approaches in HIV settings.

## Figures and Tables

**Table 1:** Characteristics of n = 23 providers who work at HIV clinics in Birmingham, AL and Pittsburgh, PA

Characteristic	Frequency (Percentage)
**Race/Ethnicity**	
White; Non-Hispanic	14 (63.7)
Black or African American	5 (22.7)
Hispanic/Latinx	1 (4.6)
Asian	1 (4.6)
More than one race	2 (9.1)
**Gender**	
Cisgender man	4 (18.2)
Cisgender woman	18 (81.8)
**Job Title**	
Pharmacist	1 (4.5)
Social Services Provider	4 (18.2)
Medical Services Provider	12 (54.5)
Administrative Services	5 (22.7)
**Number of Years Practicing Overall**	
0–5	6 (27.3)
6–10	4 (18.2)
11–15	2 (9.1)
16–20	5 (22.7)
20+	5 (22.7)
**Number of Years Working with PWH**	
0–5	8 (36.4)
6–10	7 (31.8)
11–15	2 (9.1)
16–20	2 (9.1)
20+	3 (13.6)

## Abbreviations

AL: Alabama
ART: Antiretroviral therapy
MOUD: Medication for opioid use disorder
PA: Pennsylvania
PWH: People with HIV
PWUD: People who use drugs
SSP: Syringe service program
SUD: Substance use disorder
